# Genomic Insights on Variation Underlying Capsule Expression in Meningococcal Carriage Isolates From University Students, United States, 2015–2016

**DOI:** 10.3389/fmicb.2022.815044

**Published:** 2022-02-17

**Authors:** Melissa J. Whaley, Jeni T. Vuong, Nadav Topaz, How-Yi Chang, Jennifer Dolan Thomas, Laurel T. Jenkins, Fang Hu, Susanna Schmink, Evelene Steward-Clark, Marsenia Mathis, Lorraine D. Rodriguez-Rivera, Adam C. Retchless, Sandeep J. Joseph, Alexander Chen, Anna M. Acosta, Lucy McNamara, Heidi M. Soeters, Sarah Mbaeyi, Henju Marjuki, Xin Wang

**Affiliations:** ^1^Meningitis and Vaccine Preventable Diseases Branch, Division of Bacterial Diseases, National Center for Immunization and Respiratory Diseases, Coordinating Center for Infectious Diseases, Centers for Disease Control and Prevention, Atlanta, GA, United States; ^2^CDC Foundation Field Employee assigned to the Meningitis and Vaccine Preventable Diseases Branch, Division of Bacterial Diseases, National Center for Immunization and Respiratory Diseases, Centers for Disease Control and Prevention, Atlanta, GA, United States; ^3^IHRC Inc., Contractor to Meningitis and Vaccine Preventable Diseases Branch, National Center for Immunization and Respiratory Diseases, Centers for Disease Control and Prevention, Atlanta, GA, United States

**Keywords:** *Neisseria meningitidis*, carriage, capsule, whole genome sequencing, mutations, variation

## Abstract

In January and February 2015, *Neisseria meningitidis* serogroup B (NmB) outbreaks occurred at two universities in the United States, and mass vaccination campaigns using MenB vaccines were initiated as part of a public health response. Meningococcal carriage evaluations were conducted concurrently with vaccination campaigns at these two universities and at a third university, where no NmB outbreak occurred. Meningococcal isolates (*N* = 1,514) obtained from these evaluations were characterized for capsule biosynthesis by whole-genome sequencing (WGS). Functional capsule polysaccharide synthesis (*cps*) loci belonging to one of seven capsule genogroups (B, C, E, W, X, Y, and Z) were identified in 122 isolates (8.1%). Approximately half [732 (48.4%)] of isolates could not be genogrouped because of the lack of any serogroup-specific genes. The remaining 660 isolates (43.5%) contained serogroup-specific genes for genogroup B, C, E, W, X, Y, or Z, but had mutations in the *cps* loci. Identified mutations included frameshift or point mutations resulting in premature stop codons, missing or fragmented genes, or disruptions due to insertion elements. Despite these mutations, 49/660 isolates expressed capsule as observed with slide agglutination, whereas 45/122 isolates with functional *cps* loci did not express capsule. Neither the variable capsule expression nor the genetic variation in the *cps* locus was limited to a certain clonal complex, except for capsule null isolates (predominantly clonal complex 198). Most of the meningococcal carriage isolates collected from student populations at three US universities were non-groupable as a result of either being capsule null or containing mutations within the capsule locus. Several mutations inhibiting expression of the genes involved with the synthesis and transport of the capsule may be reversible, allowing the bacteria to switch between an encapsulated and non-encapsulated state. These findings are particularly important as carriage is an important component of the transmission cycle of the pathogen, and understanding the impact of genetic variations on the synthesis of capsule, a meningococcal vaccine target and an important virulence factor, may ultimately inform strategies for control and prevention of disease caused by this pathogen.

## Introduction

*Neisseria meningitidis* (Nm, meningococcus) is a Gram-negative bacterium that colonizes the human upper respiratory tract. Approximately 10–15% of the general population, and 35% or more of individuals living in close communities (e.g., military or university residences) carry this bacterium without experiencing any symptoms (Stephens, 1999; Oldfield et al., 2017; Soeters et al., 2017). Meningococcal carriage may provide some protection from disease as colonization elicits a mucosal antibody response (Yazdankhah and Caugant, 2004). Meningococcal transmission occurs by direct contact with respiratory secretions from carriers. While mechanisms for the progression from meningococcal carriage to disease are still not well understood, meningococci can breach the mucosal barrier and invade the bloodstream to cause invasive disease such as bacteremia, septicemia, or meningitis, resulting in death in approximately 10–15% of cases (Tzeng and Stephens, 2000; Brandtzaeg and van Deuren, 2012).

As the capsule is a major virulence factor, invasive disease is nearly always caused by meningococci expressing the capsule. Nm is divided into 12 serogroups based on structure of the capsular polysaccharide expressed. Of those, serogroups A, B, C, W, X, and Y cause the majority of invasive disease (Tzeng et al., 2016). Several vaccines are currently available to protect against disease caused by Nm, including quadrivalent meningococcal conjugate vaccines, providing protection against serogroups A, C, W, and Y, and protein-based serogroup B meningococcal (MenB) vaccines, providing protection against serogroup B strains. Non-groupable meningococci (NmNG), or meningococci that do not express capsule, are more commonly recovered from asymptomatic carriers. However, rare cases of invasive disease caused by NmNG have been reported among patients who are immunocompromised, particularly those with complement component deficiency (Ganesh et al., 2017; McNamara et al., 2019).

The meningococcal genes involved in capsule biosynthesis and transport are well characterized and mapped to a region on the chromosome called the capsule polysaccharide synthesis (*cps*) locus (Dolan-Livengood et al., 2003; Harrison et al., 2013). Region A is responsible for capsule polysaccharide synthesis, and genes in this region have been used to categorize isolates into genogroups by either real-time polymerase chain reaction (rt-PCR) or whole genome sequencing (WGS) (Mothershed et al., 2004; World Health Organization, 2011; Marjuki et al., 2019). Regions A, B, and C are required for expression of the serogroup-specific capsule (Harrison et al., 2013). The reduction or loss of meningococcal capsule expression, as a result of phase variation, loss of the entire *cps* operon (capsule null, *cnl*), and/or incorporation of insertion sequences elements (ISEs) within the *cps* locus, may prevent recognition by the host adaptive immune response and potentially result in polysaccharide-based vaccine escape (Dolan-Livengood et al., 2003; Tzeng et al., 2016).

In 2015, meningococcal serogroup B outbreaks occurred at two universities in Oregon (OR) and Rhode Island (RI) in the United States (US). Mass campaigns with a meningococcal serogroup B vaccine were implemented to help control the outbreaks. In conjunction with these campaigns, cross-sectional meningococcal carriage evaluations were conducted at both universities as well as at a third university in RI, where no Nm cases were reported. Most of the carriage isolates collected were non-groupable by rt-PCR and slide agglutination serogrouping (SASG) (McNamara et al., 2017; Soeters et al., 2017; Breakwell et al., 2018). We further characterized these carriage isolates *via* WGS and analyzed the *cps* loci to better understand the possible mechanisms of non-groupability, the potential reversibility of capsule expression, and the likelihood of certain carried isolates causing disease.

## Materials and Methods

### Carriage Isolate Collection

From 10 cross-sectional meningococcal carriage evaluation rounds conducted between February 2015 and May 2016 at the three universities in OR and RI, 8,905 oropharyngeal swabs were collected. A total of 1,514 Nm isolates were obtained from 1,301 unique individuals. Carriage rates were stable between evaluation rounds, ranging between 11 and 24% (McNamara et al., 2017; Soeters et al., 2017; Breakwell et al., 2018). As carriage rates overall and specific to genogroup B were similar among the universities (McNamara et al., 2017; Breakwell et al., 2018), data were aggregated. Carriage evaluation study design and methods have been previously described (McNamara et al., 2017; Soeters et al., 2017; Breakwell et al., 2018). Single colony per participant was selected and subcultured for long-term storage (McNamara et al., 2017; Soeters et al., 2017; Breakwell et al., 2018). All isolates were identified as Nm by Gram stain (BD BBL), oxidase test (Hardy Diagnostics, Santa Maria, CA, United States), *sodC* rt-PCR (Dolan Thomas et al., 2011), and API NH tests (bioMérieux, Durham, NC, United States) (McNamara et al., 2017; Soeters et al., 2017; Breakwell et al., 2018). Isolates were plated once from frozen stocks for further characterization. This evaluation was determined to be public health research, rather than human subjects research; as such, review by the Centers for Disease Control and Prevention (CDC) Institutional Review Board was not required.

### Characterization of *Neisseria meningitidis* Capsule Polysaccharide Synthesis Genes by Whole Genome Sequencing

For sequencing on Illumina platforms (HiSeq2500 or MiSeq; San Diego, CA, United States), Nm genomic DNA was prepared with the 5 Prime ArchivePure DNA Purification kit (Gaithersburg, MD, United States). Genomic DNA was further prepared for Illumina sequencing, as previously described, through mechanical shearing and with the dual-index NEBNext Ultra DNA library preparation kits (New England Biolabs Inc., Ipswich, MA, United States) and AMPure XP kit (Beckman Coulter Inc., Indianapolis, IN, United States) (Retchless et al., 2016). Each isolate’s final library was loaded on 250-bp pair-end sequencing kits. Illumina reads were trimmed with cutadapt (Martin, 2011) and assembled with SPAdes 3.7.0 (Bankevich et al., 2012). Multilocus sequence typing (MLST) was performed from genome assemblies as previously described (Retchless et al., 2016) to provide sequence type (ST) and clonal complex (CC). The sequence reads for all isolates used in this analysis are available under NCBI BioProject PRJNA533315.

Capsule genes were identified in each assembly as previously described (Marjuki et al., 2019), using allele sequences from the PubMLST *Neisseria* database and Insertion-Sequence (IS) element sequences from the ISFinder database (Altschul et al., 1997; Siguier et al., 2006; Jolley et al., 2018). Capsule polysaccharide biosynthesis and transport genes were identified for each serogroup, specifically in regions A, B, C, and E of the *cps* locus (Harrison et al., 2013). The presence of at least one serogroup-specific gene allowed for genogroup assignment. Isolates lacking any serogroup-specific gene were referred to as genogroup non-groupable. Genogroup non-groupable isolates were further classified by the presence of some *cps* genes (NG_undetermined) or absence of the full *cps* locus (NG_*cnl*). The *cps* locus was examined for completeness and for genetic mutations, such as frameshift or point mutations resulting in premature stop codons (referred to as internal stops), missing or fragmented genes, or disruptions due to insertion elements (Marjuki et al., 2019). For this analysis, phase variation was identified from allele sequences in their phase variable off form as described by PubMLST; these allele sequences contain frameshift mutations arising from length variation from mononucleotide repeats in the sequence. Isolates that did not contain any mutations in their *cps* loci and had all expected capsule genes for the serogroup were designed as containing a “functional *cps* locus” indicating that they are predicted to express the capsule.

### Phenotypic Characterization of *Neisseria meningitidis* Capsule

Capsule type and expression in Nm carriage isolates were determined by SASG (World Health Organization, 2011; Breakwell et al., 2018). Isolates were considered groupable if agglutination of 3–4 + intensity was only observed for a single antiserum, except for serogroup Z isolates, which may agglutinate for both E/Z’ and Z antisera as described in the product insert (DIFCO, Franklin Lakes, NJ, United States). No agglutination, weak agglutination (1–2 +), polyagglutination (except for serogroup Z isolates), and autoagglutination (as observed in saline) were interpreted as non-groupable. We compared *cps* locus nucleotide sequences from phenotypically non-groupable vs. groupable Nm isolates to assess the association between specific genetic changes and capsule expression. When isolates with identical mutations produced different SASG results or when isolates with functional *cps* loci did not agglutinate, two or more operators performed repeat SASG testing with the same lot of antisera, if available.

## Results

### Characterization of *Neisseria meningitidis* Capsule by Whole Genome Sequencing and Slide Agglutination Serogrouping

Of the 1,514 meningococcal isolates recovered from the three universities, 782 (51.7%) contained serogroup-specific genes and underwent genogrouping; 732 isolates (48.3%) were NG_*cnl* or NG_undetermined by WGS. A total of 740 isolates (48.9%) had mutations in the *cps* locus, 122 (8.1%) isolates had a functional *cps* locus, and 652 (43%) isolates were capsule null (Table 1).

**TABLE 1:** Genogrouping of Nm carriage isolates from students at three US universities, 2015–2016.

Genogroup by WGS	No. isolates	No. isolates with functional *cps* loci	No. isolates predicted to not express capsule^a^
B	251	56	195
E	426	43	383
Y	36	15	21
C	31	5	26
W	4	1	3
X	12	1	11
Z	22	1	21
NG_undetermined^b^	80	NA	80
NG_*cnl*	652	NA	652
**Total**	1,514	122	1,392

*cnl, capsule null; cps, capsule polysaccharide synthesis; NA, not applicable; NG, non-groupable; Nm, Neisseria meningitidis; No., number.*

*^a^Includes isolates harboring capsule mutations including frameshift, phase variation, point mutations, gene deletions, missing genes, or gene disruptions due to insertion elements, as well as isolates which were capsule null.*

*^b^Isolates containing portions of the cps loci but lacking serogroup-specific genes.*

Of the 782 genogroupable isolates, the most common genogroups were E [426/1,514 (28.1%)] and B [251/1,514 (16.6%)]. Genogroups C, W, X, Y, and Z were also detected [4–36 isolates out of 1,514 (0.3–2.4%)]. Genogroup A was not detected in this collection. Most of these isolates (660/782) harbored mutations in the capsule biosynthesis region that may inhibit capsule expression. These mutations included phase variable off, frameshift or point mutations resulting in premature stop codons, missing or fragmented genes, and/or disruptions due to insertion elements. The remaining 122 isolates had functional capsule loci for the following genogroups: B [56 (45.9%)], E [43 (35.2%)], Y [15 (12.3%)], C [5 (4.1%)], W [1 (0.8%)], X [1 (0.8%)], and Z [1 (0.8%)] (Table 1). By SASG, the majority of isolates [1,388/1,514 (91.7%)] were non-groupable (McNamara et al., 2017; Soeters et al., 2017; Breakwell et al., 2018), and 126 isolates agglutinated with a single antiserum, or two antisera in the case of some serogroup Z isolates. Most of these isolates were either serogroup B [65/126 (51.6%)] or E [53/126 (42.1%)]; serogroups C (*n* = 1), W (*n* = 1), Y (*n* = 5) and Z (*n* = 1) represented the remaining serogroupable isolates (6.3%).

### Genetic Mutations Within the Capsule Polysaccharide Synthesis Loci

The predominant *cps* mutations were assessed among the 740 isolates for each genogroup and the NG_undetermined group (Supplementary Table 1). Phase variable off, which commonly presented as frameshifts arising from length variation in simple sequence repeats within the *cps* locus, was found most commonly among genogroup B isolates [84/195 (43.1%)], in comparison to other genogroups [1/26 (3.8%), C; 9/409 (2.2%), E; 0% for all other genogroups]. Deletions of capsule biosynthesis and transport genes were observed among the genogroup C isolates with mutations; 14 of the 26 (53.8%) were missing *cssA*, *cssB*, *cssC*, *ctrA*, *ctrB*, *ctrC*, and *ctrD*. The disruption of a capsule biosynthesis gene by IS*1301* in combination with missing genes or internal stops in other capsule biosynthesis genes was detected in more than a third of genogroup E [146/383 (38.1%)] isolates and most of genogroup Z [19/21 (90.5%)] isolates with mutations. The specific capsule biosynthesis genes disrupted by ISEs for all genogroups are indicated in Supplementary Table 2. Internal stops within the capsule biosynthesis genes (*cssC*, *csy*, or *cssA*) were commonly found among genogroup Y isolates [12/21 (57.1%)]; some of these genogroup Y isolates also contained either a fragmented *csy* (2/12) or a *cssA* disrupted by IS*1301* (3/12). All mutations (internal stops or fragmented genes) in the 11 genogroup X isolates affected *csxA*, whereas the three genogroup W isolates with mutations either had an ISE in *cssA* or had multiple biosynthesis genes missing along with an internal stop in the polymerase gene. In addition to lacking serogroup-specific genes, 70/80 (87.5%) genogroup NG_undetermined isolates contained deletions of the biosynthesis genes and portions of the capsule translocation genes in region B.

### The Capsule Polysaccharide Synthesis Genes in *Neisseria meningitidis* Isolates With Discrepant Slide Agglutination Serogrouping and Whole Genome Sequencing Results

Isolates with functional *cps* loci are thought to express capsule polysaccharide (Marjuki et al., 2019). A total of 126 isolates expressed capsule polysaccharide as determined by SASG. Among these 126 isolates, 77 were identified to have a functional *cps* locus by WGS [42 (54.5%) for B, 29 (37.6%) for E, 3 (3.9%) for Y, 1 (1.3%) for C, 1 (1.3%) for W, and 1 (1.3%) for Z]. The remaining 49 isolates had various mutations identified in their *cps* loci [23 (46.9%) for B, 24 (49.0%) for E, and 2 (4.1%) for Y]. Internal stops, ISEs, and phase variable off were commonly found among these isolates (Table 2). Of the 23 serogroup B isolates, 20 contained *csb* alleles that were determined to be in the phase variable off state based on their genome sequence. Most of the serogroup E isolates contained internal stop codons in either *cseA* or *cseD* genes. One serogroup Y isolate had an internal stop in capsule biosynthesis gene, whereas one had an ISE.

**TABLE 2:** The *cps* mutations in Nm carriage isolates with discrepant capsule typing results.

Serogroup by SASG (No.)	WGS analysis	No. (%)
B (23)	Internal stop in *csb*	1 (4.3%)
	Internal stop in *cssA*	1 (4.3%)
	Internal stop in *cssA*, phase variable off^a^ in *csb*	1 (4.3%)
	Internal stop in *ctrE*	1 (4.3%)
	Phase variable off in *csb*	19 (82.6%)
E (24)	*cseD* disrupted by IS*1301*	3 (12.5%)
	Internal stop in *cseA*	6 (25%)
	Internal stop in *cseD*	10 (41.7%)
	Missing *cseA*, missing *cseB*, missing *cseC*, missing *cseD*	1 (4.1%)
	*cseG* fragmented (31.89% coverage), *ctrA* fragmented (20.98% coverage), internal stop in *cseG*, missing *cseA*, missing *cseB*, missing *cseC*, missing *cseD*, missing *cseE*	1 (4.1%)
	Internal stop in *cseC*, internal stop in *ctrA*	1 (4.1%)
	Internal stop in *cseD*, internal stop in *cseG*	2 (8.3%)
Y (2)	Internal stop in *cssA*	1 (50%)
	*cssC* disrupted by IS*1655*	1 (50%)

*Nm, Neisseria meningitidis; No., number of isolates; SASG, slide agglutination serogrouping; WGS, whole genome sequencing.*

*^a^Defined as frameshifts arising from length variation in mononucleotide repeats.*

The mutations identified among the 47 serogroupable isolates were also detected among non-serogroupable isolates (Supplementary Table 3), indicating that the same mutation may or may not prevent capsule expression as determined by SASG. One of the two (50%) genogroup Y isolates agglutinated with Y antisera when both isolates possessed an internal stop in the same location of the *cssA*. One of 24 (4.2%) genogroup B isolates with an internal stop in the same location of *cssA* expressed a B capsule, as well as 19 of 64 (30%) genogroup B isolates with *csb* alleles presumably in the phase variable off state. Six of 48 (11.1%) genogroup E isolates containing a premature internal stop in *cseA* and 13/16 (81.3%) of genogroup E isolates with premature internal stop in *cseD* agglutinated with the E antisera. Among 122 isolates containing a functional *cps* locus, 45/122 (36.9%) isolates were non-groupable by SASG (Table 1 and Supplementary Table 1), including genogroups B [14 (31.1%)], E [14 (31.1%)], Y [12 (26.7%)], C [4 (8.9%)], and X [1 (2.2%)]. As previously described, the remaining 77 isolates with functional *cps* loci were serogroupable.

### Association Between Genogroup and Clonal Complex

**FIGURE 1:**
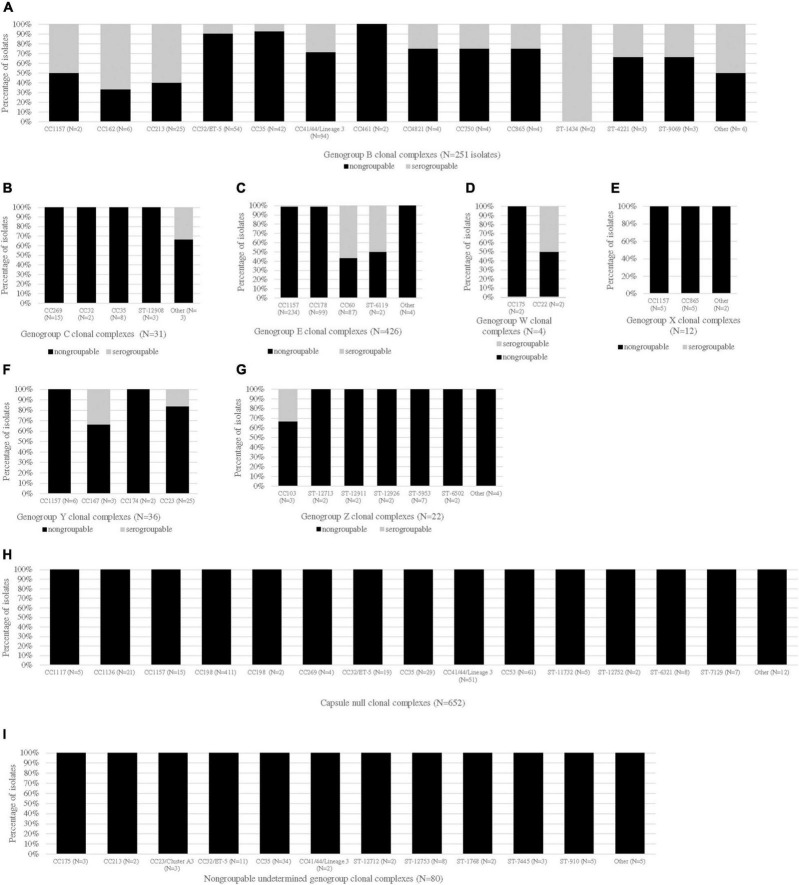
CC distribution of Nm carriage isolates by genogroup and serogroup. The proportions of serogroupable (shown in gray) and non-serogroupable (shown in black) meningococcal isolates were presented for each genogroup-specific clonal complex (CC): genogroup B **(A)**, genogroup C **(B)**, genogroup E **(C)**, genogroup W **(D)**, genogroup X **(E)**, genogroup Y **(F)**, genogroup Z **(G)**, capsule null **(H)**, and non-groupable undetermined genogroup **(I)**. If a CC was unassigned, the sequence type (ST) was provided. The “other” category represents the genogroup-specific CCs that contained only one isolate. The serogroupable isolates belonging to the “other” category were as follows: serogroup B: CC174, ST-11860 and ST-948; and serogroup C: ST-12922.

The CC distribution by genogroup is shown in Figure 1. Most CCs were unique to a genogroup, except for CC1157, CC162, CC167, CC174, CC175, CC178, CC213, CC23, CC269, CC32, CC35, CC44/41, CC60, CC865, and unassigned CC (ST-5953). The most diverse CC by genogroup, CC1157, was the predominant CC for genogroup E isolates [232/426 (54.5%)] and was one of the most common CCs for genogroup X isolates [5/12 (41.7%)]. The other common genogroup X CC was CC865 [5/12 (41.7%)]. The predominant CCs for the other genogroups were as follows: CC41/44 for genogroup B [94/251 (37.5%)], CC269 for genogroup C [15/31 (48.3%)], CC23 for genogroup Y [21/36 (58.3%)], unassigned CC (ST-5953) for genogroup Z [7/22 (31.8%)], CC35 for genogroup NG_undetermined isolates [34/80 (42.5%)], and CC198 for genogroup NG_*cnl* isolates [413/652 (63.3%)]. Genogroup W isolates belonged to either CC175 or CC22.

## Discussion

In this study, we characterized the capsule type and expression of Nm isolates obtained from carriage evaluations at three US universities in 2015–2016 and identified genetic variations within the *cps* loci of these isolates. Most of these isolates were genetically non-groupable because of lack of serogroup-specific genes (NG_undetermined) or the whole *cps* locus (NG_*cnl*), followed by genogroups E and B. A majority of these isolates contained genetic mutations such as phase variable off, frameshift or point mutations, missing or fragmented genes, and/or disruptions due to insertion elements within the *cps* loci. A functional *cps* locus was detected only in a very small proportion of Nm carriage isolates.

While genogroup composition among Nm carriage isolates is similar to other carriage studies, E was the most common genogroup among genogroupable isolates, representing 26% of the collection. In contrast, carriage evaluations conducted at universities in Europe and South America identified genogroup B as the most common genogroup; genogroup E isolates were between 2 and 9% of their collection (Gasparini et al., 2014; Rodriguez et al., 2014; Cassio de Moraes et al., 2015; Jeppesen et al., 2015; Díaz et al., 2016; Tryfinopoulou et al., 2016; Moura et al., 2017; van Ravenhorst et al., 2017; Oldfield et al., 2018). In earlier US carriage evaluations, genogroup E carriage was 10% among high school students (Harrison et al., 2015). The higher proportion of genogroup E isolates may be specific to these US university populations at the time of collection. In a review, meningococcal carriage differed in serogroup distribution within and between European countries, across age groups, and over time (Soriano-Gabarró et al., 2011). Serological methods, such as SASG, were more often used than molecular methods in earlier carriage evaluations (Soriano-Gabarró et al., 2011). The carriage of genogroup E and other rare genogroups may be underestimated in earlier carriage studies as a result of the limited availability of antiserum for these serogroups (Soriano-Gabarró et al., 2011; Harrison et al., 2013; Rodrigues et al., 2015). Genogroup C isolates were rarely detected in carriage evaluations conducted during a time when NmC conjugate vaccines were widely available (Gasparini et al., 2014; Harrison et al., 2015; Moreno et al., 2015; Rodrigues et al., 2015; Tryfinopoulou et al., 2016; Moura et al., 2017; McMillan et al., 2019). Consistently, genogroup C represents only a small proportion of carriage isolates in our collection, which may be due to the routine recommendations for use of meningococcal ACWY vaccines among adolescents (Mbaeyi et al., 2020).

Predominant *cps* mutations for each genogroup were assessed to understand their impact on capsule expression. While some genetic mutations result in irreversible loss of capsule expression, some mutations, such as frameshifts and point mutations that were commonly found in the capsule biosynthesis genes of genogroup B and E isolates in our collection, may serve as an on–off switch to regulate capsule expression under different conditions. This on–off switch can down-regulate capsule expression to allow Nm colonization during carriage and up-regulate it to allow progression to disease (Hammerschmidt et al., 1996; Weber et al., 2006; Tzeng et al., 2016) by introducing or removing premature internal stops. In addition, we also observed that several genogroup B isolates with *csb* in the phase variable off state still expressed a B capsule, suggesting phase variable status may change during isolation and culturing of Nm (Lucidarme et al., 2013; Siena et al., 2016). Capsule expression has also been detected by phenotypic methods for isolates with ISEs in *cssA*, *cssE*, *cssF*, or *csb* in earlier studies (Hammerschmidt et al., 1996; Weber et al., 2006; Germinario et al., 2010; Kugelberg et al., 2010; Loh et al., 2013; Jones et al., 2016; Tzeng et al., 2016). In this study, agglutination was observed among genogroup E isolates containing internal stops in *cseD*, suggesting that the disruption *cseD* did not prevent capsule expression. Another explanation may be that the function of *cseD*, lipopolysaccharide–KDO synthesis, is complemented elsewhere in the genome (Harrison et al., 2013; Tzeng et al., 2016). An unlinked gene complementation may also explain agglutination for genogroup B and E isolates with internal stops in the biosynthesis genes. One of three genogroup B isolates with an internal stop in *csb* agglutinated, which was also observed for three invasive serogroup B isolates in a previous study (Marjuki et al., 2019). It warrants further investigation whether the different position of the stop codon(s) may possibly have permitted translation of gene products among these isolates, resulting in some capsule expression.

A subset of isolates belonging to genogroups B and Y with functional *cps* loci did not agglutinate with the capsule-specific antisera (non-groupable phenotype). Similar lack of agglutination has been observed in previous studies evaluating both invasive and carriage isolates among university students and general populations (Germinario et al., 2010; Gasparini et al., 2014; Jones et al., 2016; van Ravenhorst et al., 2017; Marjuki et al., 2019). These isolates may have had a biosynthesis gene in the phase variable on state when sequenced and changed to off state when tested for agglutination, as a result of subculturing a single colony (Lucidarme et al., 2013; Siena et al., 2016). To assess the impact of culture preparation on phase variation–mediated capsule expression, we are sequencing multiple colonies from a single stock under the same conditions described in the methods (Siena et al., 2016) and testing their capsule expression using agglutination and other methods. We are also examining non-capsule genes to see if they have an impact on capsule expression. These studies in progress may help address the discrepancies we observed. Other reasons for this observation could be that the expression of capsule was at a level so low that was undetectable by SASG (Mothershed et al., 2004; Soriano-Gabarró et al., 2011; Jeppesen et al., 2015; Gilca et al., 2018). SASG testing was repeated on these isolates by different operators and yielded similar results. Although SASG is a common method for serogrouping Nm with relatively good sensitivity and specificity (van der Ende et al., 1995), we have noted differences between or within lots of antiserum, which unavoidably affect the performance of this test and lead to discrepancies when compared with other test results such as WGS. We have conducted preliminary testing to investigate other phenotypic tests, but so far, discrepancies have not resolved by other methods; this is in agreement with Jones et al. (2016), who assessed specificity and sensitivity of agglutination and other phenotypic tests (flow cytometry, live-cell phenotypic assay, and dot blotting) in comparison to WGS. Others also have observed that, even with the use of other phenotypic assays (Ouchterlony and live-cell phenotypic assay), some genogroup B carriage isolates with functional *cps* loci remained non-groupable (van Ravenhorst et al., 2017; Gilca et al., 2018). Genes adjacent to the *cps* locus and non-coding regions outside of the *cps* locus may influence capsule expression (Swartley et al., 1997). The inactivation of KpsF, encoded by a gene outside of the *cps* locus, was shown to reduce capsule expression (Tzeng et al., 2016). However, the carriage isolates studied here contained an intact *kpsF* (data not shown), but this does not rule out the possibility of the gene expression being inactivated. Further analysis of non-*cps* genes and non-coding regions is warranted to better understand the non-groupability of isolates with functional *cps* loci.

In comparison to other carriage studies (Soriano-Gabarró et al., 2011; Gasparini et al., 2014; Read et al., 2014; Rodriguez et al., 2014; Cassio de Moraes et al., 2015; Jeppesen et al., 2015; Moreno et al., 2015; Díaz et al., 2016; Tryfinopoulou et al., 2016; Moura et al., 2017; Oldfield et al., 2017, 2018; Tekin et al., 2017; van Ravenhorst et al., 2017; Gilca et al., 2018; Terranova et al., 2018; McMillan et al., 2019), similar CC distributions among genogroups were observed. Capsule null isolates commonly belonged to CC198 and rarely belonged to CCs associated with invasive disease (Gasparini et al., 2014; Rodriguez et al., 2014; Cassio de Moraes et al., 2015; Moreno et al., 2015; Rodrigues et al., 2015; Tryfinopoulou et al., 2016; Moura et al., 2017; Oldfield et al., 2018; McMillan et al., 2019). The majority of genogroup Y isolates from our collection belonged to CC23, similar to the US carriage evaluation among high school students (Harrison et al., 2015) and to university carriage studies in Europe (Gasparini et al., 2014; Rodrigues et al., 2015; Oldfield et al., 2018), South America (Moreno et al., 2015; Moura et al., 2017), and Australia (McMillan et al., 2019). Genogroup B isolates predominantly belonged to CC41/44 (Soriano-Gabarró et al., 2011; Rodriguez et al., 2014; Jeppesen et al., 2015; Moreno et al., 2015; Rodrigues et al., 2015; Tryfinopoulou et al., 2016; Tekin et al., 2017; Gilca et al., 2018; McMillan et al., 2019). Genogroup C in this evaluation belonged to CCs commonly associated with *N. meningitidis* serogroup B disease (CC269, CC32, CC35, and CC41/44). Genogroup C isolates belonging to CC41/44 and CC35 were also detected among university carriage isolates collected in Brazil (Cassio de Moraes et al., 2015; Moura et al., 2017) and Colombia (Moreno et al., 2015). CC269 genogroup C isolates appear to be uniquely detected in our collection.

## Data Availability Statement

The datasets presented in this study can be found in online repositories. The names of the repository/repositories and accession number(s) can be found below: https://www.ncbi.nlm.nih.gov/, BioProject PRJNA533315.

## Author Contributions

MW, JV, NT, HM, and XW conceptualized, performed the analysis, and wrote the manuscript. HC, JT, LJ, FH, SS, ES-C, MM, and LR-R involved in testing of the samples. AA, LM, HS, and SM involved in managing and supervising the carriage studies. AR, SJ, and AC involved in data analysis, interpretation, and data management. XW supervised the project and the wrote of the manuscript. All authors reviewed the manuscript and provided critical feedback.

## Conflict of Interest

HC, LJ, FH, MM, LR-R, and SJ were employed by the IHRC Inc. The remaining authors declare that the research was conducted in the absence of any commercial or financial relationships that could be construed as a potential conflict of interest.

## Publisher’s Note

All claims expressed in this article are solely those of the authors and do not necessarily represent those of their affiliated organizations, or those of the publisher, the editors and the reviewers. Any product that may be evaluated in this article, or claim that may be made by its manufacturer, is not guaranteed or endorsed by the publisher.
